# Perampanel, a potent AMPA receptor antagonist, protects against tetramethylenedisulfotetramine-induced seizures and lethality in mice: comparison with diazepam

**DOI:** 10.1007/s00204-021-03053-9

**Published:** 2021-04-29

**Authors:** Dorota Zolkowska, Ashish Dhir, Michael A. Rogawski

**Affiliations:** 1 Department of Neurology, School of Medicine, University of California, Davis, Sacramento, CA 95817 USA; 2 Department of Pharmacology, School of Medicine, University of California, Davis, Sacramento, CA 95817 USA

**Keywords:** Tetramethylenedisulfotetramine, Perampanel, Diazepam, Seizure, AMPA receptor antagonist

## Abstract

Tetramethylenedisulfotetramine (TETS), a noncompetitive GABA_A_ receptor antagonist, is a potent, highly lethal convulsant that is considered to be a chemical threat agent. Here, we assessed the ability of the AMPA receptor antagonist perampanel to protect against TETS-induced seizures and lethality in mice when administered before or after treatment with the toxicant. For comparison, we conducted parallel testing with diazepam, which is a first-line treatment for chemically induced seizures in humans. Pre-treatment of mice with either perampanel (1–4 mg/kg, i.p.) or diazepam (1–5 mg/kg, i.p.) conferred protection in a dose-dependent fashion against tonic seizures and lethality following a dose of TETS (0.2 mg/kg, i.p.) that rapidly induces seizures and death. The ED_50_ values for protection against mortality were 1.6 mg/kg for perampanel and 2.1 mg/kg for diazepam. Clonic seizures were unaffected by perampanel and only prevented in a minority of animals by high-dose diazepam. Neither treatment prevented myoclonic body twitches. Perampanel and diazepam also conferred protection against tonic seizures and lethality when administered 15 min following a 0.14 mg/kg, i.p., dose of TETS and 5 min following a 0.2 mg/kg, i.p., dose of TETS. Both posttreatments were highly potent at reducing tonic seizures and lethality in animals exposed to the lower dose of TETS whereas greater doses of both treatments were required in animals exposed to the larger dose of TETS. Neither treatment was as effective suppressing clonic seizures. In an experiment where 0.4 mg/kg TETS was administered by oral gavage and the treatment drugs were administered 5 min later, perampanel only partially protected against lethality whereas diazepam produced nearly complete protection. We conclude that perampanel and diazepam protect against TETS-induced tonic seizures and lethality but have less impact on clonic seizures. Both drugs could have utility in the treatment of TETS intoxication but neither eliminates all seizure activity.

## Introduction

Tetramethylenedisulfotetramine (TETS) is a potent convulsant poison (Zolkowska et al. 2012; Rice et al. 2017; Lauková et al. 2020) considered to be a chemical threat agent by the National Institutes of Health (Jett and Spriggs 2020) and other health authorities (Patocka et al. 2018). TETS is believed to induce lethal seizures by blocking brain GABA_A_ receptors (Bowery et al. 1975; Large 1975; Dray 1975; Roberts et al. 1981; Nik et al. 2017; Pressly et al. 2021). No specific antidote for TETS intoxication is known. However, recent studies indicate that positive modulators of GABA_A_ receptors, such as diazepam, can prolong the time to onset of TETS-induced convulsions and reduce lethality (Shakarjian et al. 2012; Vito et al. 2014; Bruun et al. 2015; Moffett et al. 2019).

Inhibitors of NMDA and AMPA ionotropic glutamate receptors, which are the principal mediators of fast excitatory neurotransmission in the brain, have been extensively studied as potential seizure treatments (Löscher and Rogawski 2002). While NMDA receptor antagonists are anticonvulsant in certain in vitro and in vivo models, they can paradoxically promote seizures in other situations (Rogawski 2013). Therefore, it is of interest that NMDA antagonists, such as ketamine and dizocilpine (MK-801), may increase the frequency of TETS-induced seizures (Shakarjian et al. 2012) although when used in combination with positive modulators of GABA_A_ receptors, these deleterious actions are not observed (Shakarjian et al. 2015). Unlike NMDA receptor antagonists, which have not proven useful in the clinical treatment of seizures in people with epilepsy, AMPA receptors are a validated clinical target for epilepsy treatment. Perampanel [2-(2-oxo-1-phenyl-5-pyridin-2-yl-1,2-dihydropyridin-3-yl)benzonitrile; E2007], a potent noncompetitive AMPA receptor antagonist, has been shown to reduce the frequency of focal-onset seizures and primary generalized tonic seizures in people with epilepsy (Steinhoff 2014). In in vitro studies, perampanel selectively inhibits AMPA receptor-induced synaptic excitation without affecting responses mediated by NMDA or kainate receptors (Ceolin et al. 2012; Rogawski and Hanada 2013; Chen et al. 2014). Moreover, perampanel is believed to be highly selective for AMPA receptors and is not known to interact with other anti-seizure brain targets.

As is the case for other AMPA receptor antagonists, perampanel exhibits broad-spectrum anti-seizure activity in animal models. It potently protects mice against seizures induced by the GABA_A_ receptor antagonist pentylenetetrazol (ED_50_, 0.94 mg, p.o.); it is effective against audiogenic seizures in DBA/2 mice; and it has activity in the maximal electroshock (MES) and 6 Hz tests, as well as in kindled seizures (Hanada et al. 2011). Perampanel also reduces the frequency of focal electrographic seizures in mice that exhibit spontaneous recurrent seizures following intrahippocampal injection of kainic acid (Twele et al. 2015). Finally, perampanel suppresses behavioral and electrographic seizures in the rat lithium-pilocarpine (Hanada et al. 2014) and diisopropylfluorophosphate (Dhir et al. 2020) models of status epilepticus.

Given the broad-spectrum activity of peramapanel in animal seizure models, we hypothesized that it would also be effective in treating TETS-induced seizures. In the present study, we assessed perampanel in comparison with diazepam. We first confirmed the activity of perampanel in the MES test to verify its anti-seizure efficacy and potency when administered by the parenteral route in the strain of mice used in our laboratory. With dosing and time course of action information in hand, we then demonstrated that pre-treatment with both perampanel and diazepam reduced the incidence and severity of TETS-induced tonic seizures and lethality although they did not eliminate myoclonic seizures. However, pre-treatment is not feasible in accidental or intentional poisoning. Therefore, we sought to assess the activity of the two anti-seizure drugs with post-treatment. Both were found to protect against tonic seizures and lethality when administered following TETS exposure, but in a group of animals that received a high oral dose of TETS, perampanel failed to perform as well as diazepam.

## Materials and methods

### Animals

Male NIH Swiss mice (22–30 g) were housed four per cage. Animals were kept in a vivarium under controlled laboratory conditions (temperature 22–26 °C; humidity 40–50%) with an artificial 12-h light/dark cycle and free access to food and water. Animals were allowed to acclimate to the vivarium for ≥ 5 days. The experiments were performed during the light phase of the light/dark cycle after a ≥ 30-min period of acclimation to the experimental room. Animals were maintained in facilities fully accredited by the Association for Assessment and Accreditation of Laboratory Animal Care, and all studies were performed under protocols approved by the University of California, Davis, Institutional Animal Care and Use Committee in strict compliance with the Guide for the Care and Use of Laboratory Animals of the National Research Council (National Academy Press, Washington, DC; http://www.nap.edu/readingroom/books/labrats/). Mice that survived testing were euthanized with CO_2_.

### Test substances and formulations

TETS was synthesized by a modification of the procedure of Esser et al. (1991). Solutions of TETS were made in 100% DMSO at a concentration of 1 mg/ml. Further dilutions were made in 10% DMSO in 0.9% saline. TETS was injected intraperitoneally at a dose of either 0.14 mg/kg or 0.2 mg/kg, or administered by oral gavage at a dose of 0.4 mg/kg. Perampanel was supplied by Eisai Inc. (Ibaraki Prefecture, Japan) and diazepam was purchased from Sigma-Aldrich (St Louis, MO). Perampanel and diazepam were prepared as suspensions in 1% Tween 80 in sterile saline and were administered intraperitoneally. Drug solutions for intraperitoneal or oral dosing were administered in a volume equaling 10-ml/kg body weight.

### MES seizure test

**Fig. 1:**
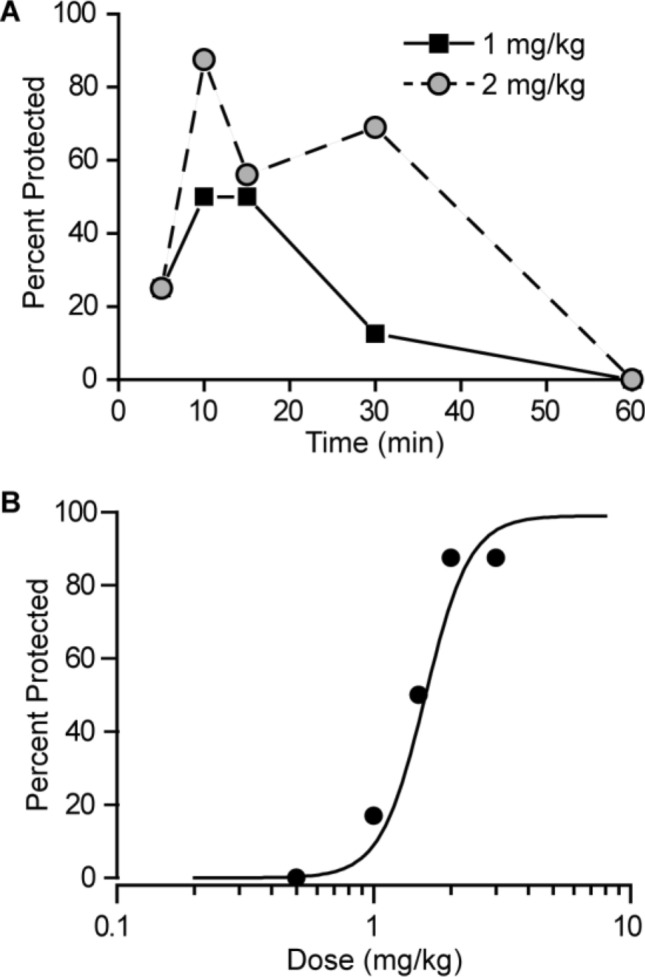
**A** Time course for protection by perampanel at the doses of 1 and 2 mg/kg, i.p., in the maximal electroshock (MES) test. The interval between perampanel injection and the electrical stimulus is plotted on the abscissa and the percentage of animals protected against seizures is plotted on the ordinate. Each point represents at least eight mice. **B** Dose–response relationship for protection by perampanel in the MES test. Perampanel was administered 10 min before the electrical stimulus. Each point represents 8–24 mice

Animals were subjected to a 0.2-s, 60-Hz electrical stimulus through corneal electrodes. The electroshock unit was adjusted to deliver a constant current of 50 mA. Animals failing to show tonic hindlimb extension were scored as protected (Kokate et al. 1994). To obtain dose–response data, vehicle or perampanel was administered i.p. 10 min before the electrical stimulus. The pre-treatment interval was based on the time of maximal effect in the time course experiment of Fig. 1A.

### Motor impairment test

Perampanel was evaluated for motor toxicity using a modification of the horizontal screen test as described previously (Kokate et al. 1994). Mice were placed on a horizontally oriented grid (consisting of parallel 1.5-mm-diameter rods situated 1 cm apart) and the grid was inverted. Animals that fell from the grid within 10 s were scored as impaired.

### Scoring of TETS-induced seizures

Animals were observed for 1 h after administration of TETS. During this period, the occurrence of myoclonic body twitches, clonic seizures (rhythmic movements of forelimbs- and/or hindlimbs for ≥ 5 s with or without loss of balance and falling), wild running, tonic seizures (loss of posture, forelimb tonic extension with hindlimb tonic contraction and/or extension that in some cases was followed by clonic movements of all limbs) and death was recorded. In experiments in which a treatment drug was administered after dosing with TETS, clonic seizures often occurred prior to administration of the treatment but was excluded in the scoring. In the assessment of the time to occurrence of clonic seizures (Fig. 4), a clonic seizure was considered terminated when all clonic seizure signs ceased completely for at least 1 min. The severity of tonic seizures was scored as either not present (0), mild (1), moderate (2), or severe (3), where “mild” indicates brief and transitory episodes of tonic forelimb extension; “moderate” indicates tonic forelimb extension with tonic hindlimb contraction that may be followed by continuous episodes of clonic seizures with loss of posture; and “severe” indicates tonic forelimb extension followed by tonic hindlimb extension. Mean convulsion severity was calculated as the sum of the individual scores divided by the number of animals. In most cases, the animals were euthanized after the 1 h observation period. In some cases, surviving animals were returned to the vivarium and their clinical status assessed at 24 h. All scoring was by the same experienced unblinded observer aided by another observer who provided verification.

### Data analysis

**Fig. 2:**
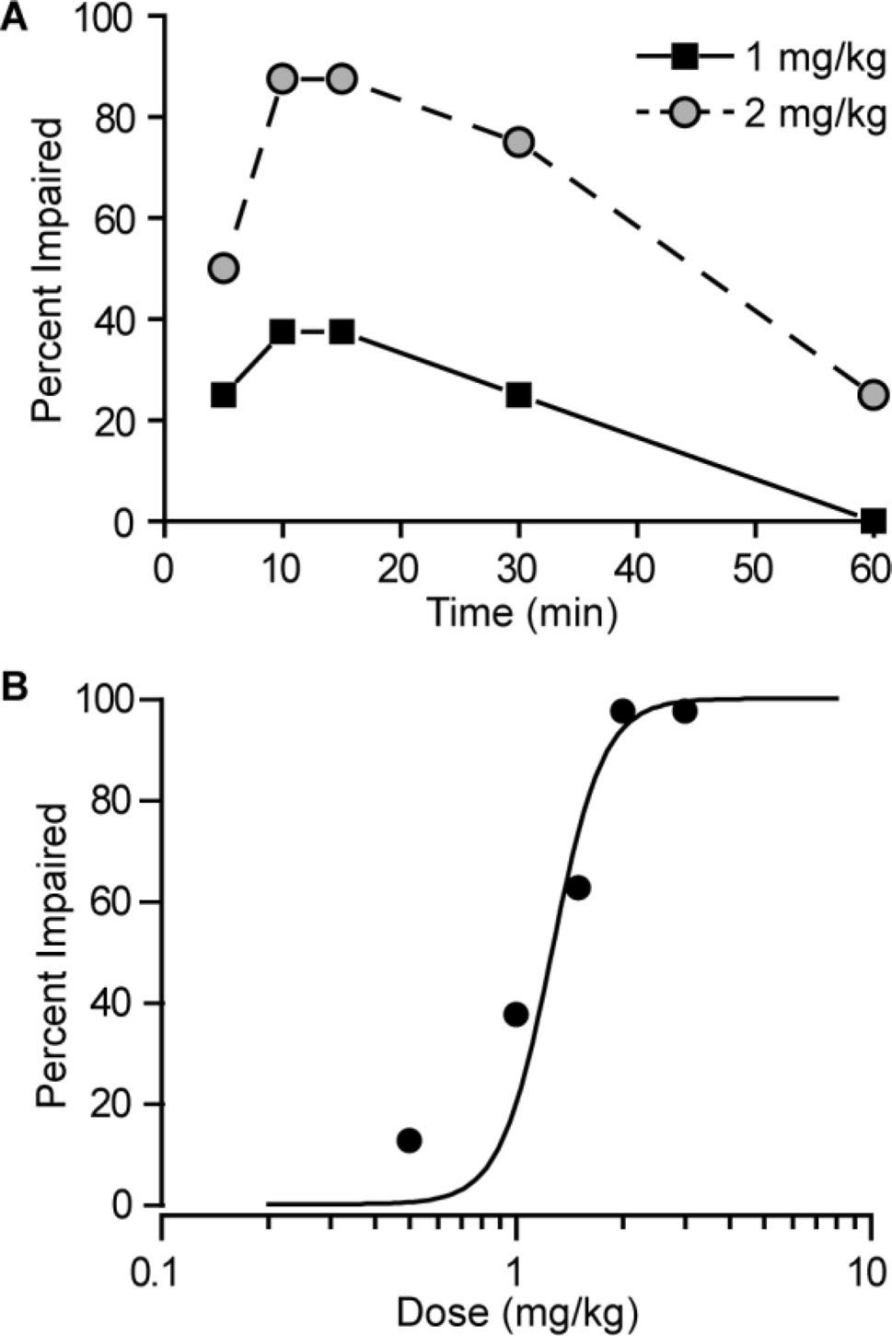
**A** Time course for induction of motor impairment by perampanel at the doses of 1 and 2 mg/kg, i.p., in the horizontal screen test. The interval between perampanel injection and testing is plotted on the abscissa and the percentage of animals exhibiting impairment is plotted on the ordinate. Each point represents at least eight mice. **B** Dose–response relationship for induction of motor impairment by perampanel. Perampanel was administered 10 min before the electrical stimulus. Each point represents 8–24 mice

To construct dose–response curves, perampanel and diazepam were tested at several doses spanning the dose (ED_50_) corresponding to protection against seizures or mortality or induction of motor impairment in 50% of animals. ED_50_ values and their corresponding standard error (SEM) and 95% confidence limits (95% CI) were determined using a probit regression model implemented as a custom-designed application in RStudio. In the analysis to determine ED_50_ values for TETS-induced tonic seizures and lethality, the slopes of the perampanel and diazepam dose–response models were constrained to be the same. Curves to dose–response data in Figs. 1, 2 were fit by eye with the Hill equation.

## Results

### MES seizure test

In confirmation of a previous report by Hanada et al. (2011), we found that pre-treatment with perampanel conferred protection in the MES seizure test. As shown in Fig. 1A, perampanel at a dose of 1 mg/kg, i.p., provided maximal protection of 50% of the animals at the 10 and 15 min time points. A smaller number of animals were protected at 30 min and protection was not present at 60 min. A higher dose of perampanel (2 mg/kg) conferred nearly complete seizure protection (85.6%) at 10 min, and substantial protection up to 30 min, as was the case with the lower dose, protection was no longer present at 60 min. We conducted a dose–response study with a 10 min pre-treatment interval. The results shown in Fig. 1B indicated that the protection occurred in a dose-dependent fashion with ED_50_ value of 1.50 mg/kg (95% CI 1.22–1.77).

### Motor impairment test

As shown in Fig. 2A, perampanel at doses of 1 and 2 mg/kg, i.p., caused motor impairment that followed a similar time course as MES seizure protection. The dose–response study shown in Fig. 2B indicated that the ED_50_ value is 1.19 mg/kg (95% CI 0.89–1.48). Animals exhibiting motor impairment did not exhibit loss of righting reflex at any of the doses tested.

### Effect of pre-treatment with peramapanel or diazepam on seizures and lethality induced by 0.2 mg/kg TETS

**Fig. 3:**
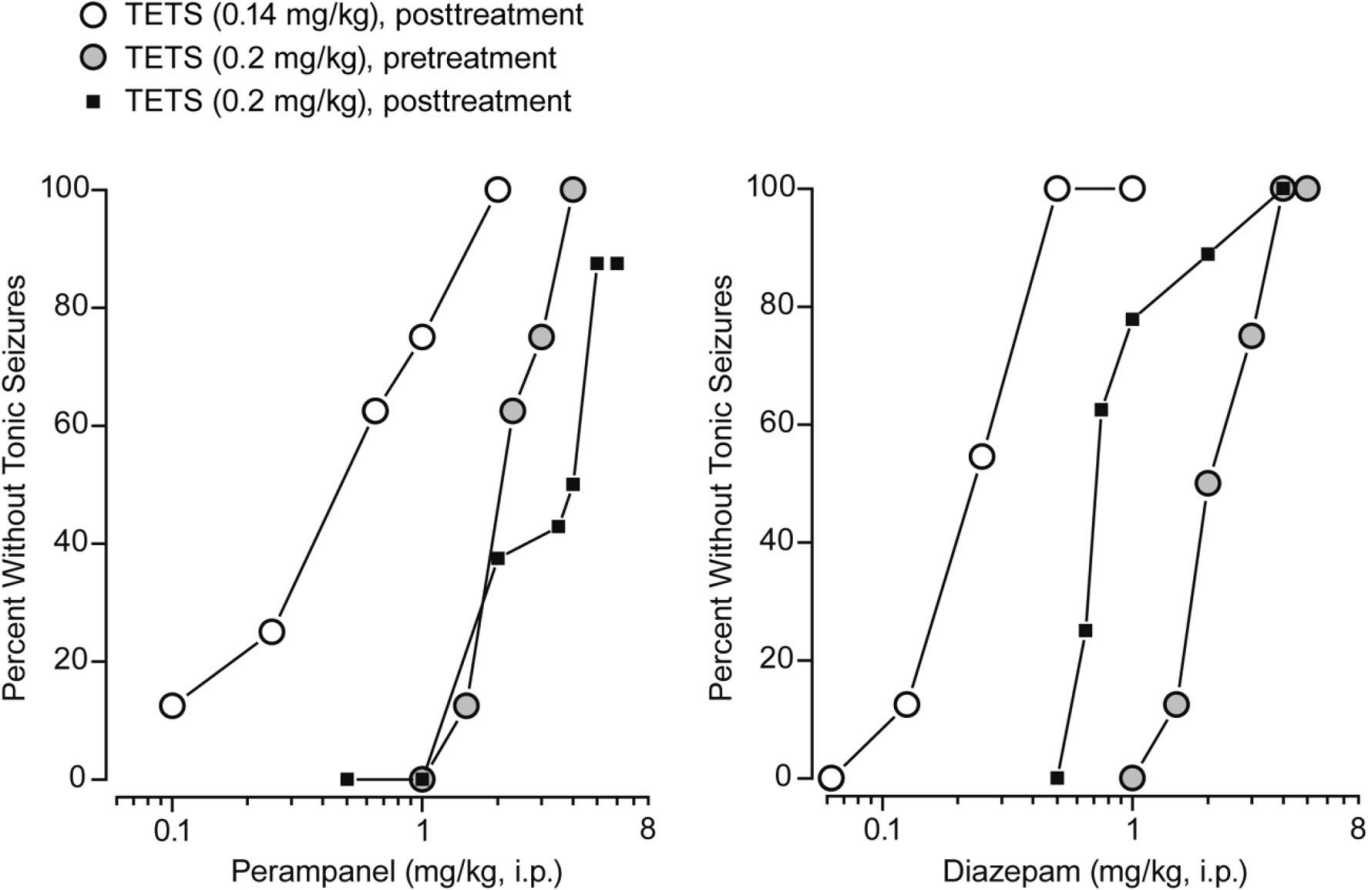
Dose–response relationships for perampanel and diazepam protection against tonic seizures in the experiment with pre-treatment prior to a 0.2 mg/kg, i.p., dose of TETS and in the experiments with post-treatment following a 0.14 mg/kg, i.p., or 0.2 mg/kg, i.p., dose of TETS. ED_50_ ± SEM and 95% CI values are presented in Table 5

Animals pre-treated with vehicle who subsequently received a 0.2 mg/kg, i.p., dose of TETS exhibited myoclonic twitches and clonic seizures, and tonic seizures of maximum severity (Table 1 and Fig. 3). These animals all expired during the 1 h observation period. Pre-treatment with perampanel (1–4 mg/kg, i.p.) 10 min before TETS or diazepam (1–5 mg/kg, i.p.) 30 min before TETS did not reduce the occurrence of myclonic body twitches or clonic seizures, except that the highest doses of diazepam did eliminate clonic seizures in some animals. These doses of perampanel and diazepam did cause a dose-dependent reduction in the incidence and severity of tonic seizures and improved survival in a dose-dependent fashion so that all animals survived when pre-treated with 4 mg/kg or higher doses of each drug. The ED_50_ values for perampanel and diazepam protection against tonic seizures and lethality are presented in Table 5.

**Table 1:** Effects of pre-treatment with i.p. perampanel 10 min before and i.p. diazepam 30 min before TETS (0.2 mg/kg, i.p.) on the incidence of seizure signs, tonic seizure severity, and the incidence of lethality during a 1 h observation period

Treatment (dose)	*N*	Myoclonic body twitches	Clonic seizures	Tonic seizures	Mean tonic seizure severity^1^	Dead 1 h
Vehicle for perampanel group (10 min pre-treatment)	8	100%	100%	100%	3.0	100%
Perampanel (1 mg/kg)	8	100%	100%	100%	2.4	75%
Perampanel (1.5 mg/kg)	8	100%	100%	87.5%	2.3	50%
Perampanel (2.3 mg/kg)	8	100%	100%	37.5%	1.0	25%
Perampanel (3 mg/kg)	8	100%	100%	25%	0.8	25%
Perampanel (4 mg/kg)	8	100%	100%	0%	0	0%
Vehicle for diazepam group (30 min pre-treatment)	8	100%	100%	100%	3.0	100%
Diazepam (1 mg/kg)	8	100%	100%	100%	3.0	100%
Diazepam (1.5 mg/kg)	8	100%	100%	87.5%	2.6	87.5%
Diazepam (2 mg/kg)	8	100%	100%	50%	1.5	50%
Diazepam (3 mg/kg)	8	100%	100%	25%	0.8	25%
Diazepam (4 mg/kg)	8	100%	87.5%	0%	0	0%
Diazepam (5 mg/kg)	8	100%	62.5%	0%	0	0%

^1^Tonic seizures were scored on a 4-point scale (0–3) where 3 is maximum severity; the values shown are the means

### Effect of post-treatment with perampanel or diazepam on seizures and lethality induced by 0.14 mg/kg or 0.2 mg/kg TETS

In cases of accidental or intentional exposure, treatments would be administered following exposure to TETS and we therefore sought to determine if perampanel is effective when administered in a post-exposure paradigm. With a 0.14 mg/kg, i.p., dose of TETS animals exhibited a delayed onset to seizure signs and survived sufficiently long to permit treatment administration 15 min after TETS exposure. As shown in Table 2 and Fig. 3, perampanel (0.1–2 mg/kg, i.p.) only minimally affected clonic seizures but did cause dose-dependent protection against tonic seizure and lethality. Diazepam (0.0625–1 mg/kg, i.p.) provided a similar dose-dependent protection. The ED_50_ values for perampanel and diazepam protection against tonic seizures and lethality are presented in Table 5.

**Table 2:** Effects of post-treatment with perampanel and diazepam i.p. 15 min following a 0.14 mg/kg, i.p., dose of TETS on the incidence of clonic and tonic seizures, tonic seizure severity, and the incidence of lethality at 1 h and 24 h

Treatment (dose)	*N*	Clonic seizures after treatment	Tonic seizures	Mean tonic seizure severity	Dead 1 h	Dead or unsalvageable 24 h
Vehicle	32	100%	90.6%	2.7	90.6%	96.9%
Perampanel (0.1 mg/kg)	8	100%	87.5%	2.6	87.5%	100%
Perampanel (0.25 mg/kg)	8	100%	75%	2.3	37.5%	50%
Perampanel (0.65 mg/kg)	8	100%	37.5%	1.1	12.5%	12.5%
Perampanel (1 mg/kg)	8	87.5%	25%	0.8	25%	25%
Perampanel (2 mg/kg)	8	87.5%	0%	0	0%	0%
Diazepam (0.0625 mg/kg)	8	100%	100%	3.0	75%	100%
Diazepam (0.125 mg/kg)	8	100%	87.5%	2.6	87.5%	87.5%
Diazepam (0.25 mg/kg)	22	95.5%	45.5%	1.4	36.4%	59.1%
Diazepam (0.5 mg/kg)	17	100%	0%	0	0%	29.4%
Diazepam (1 mg/kg)	8	62.5%	0%	0	0%	12.5%

Following administration of the higher (0.2 mg/kg) dose of TETS used in the pre-treatment experiments (see above), animals experienced the rapid occurrence of myoclonic twitches, clonic seizures, tonic seizures and death within less than 15 min, so that a briefer post-treatment interval is required. We therefore conducted experiments with a post-treatment interval of 5 min. As shown in Table 3, doses of perampanel from 0.5 mg/kg to 6 mg/kg had minimal effect on clonic seizures as in the other pre-treatment and post-treatment experiments. These higher doses of perampanel did confer dose-dependent protection from tonic seizures (Fig. 3) and death. Diazepam also provided protection against tonic seizures and lethality, and high doses inhibited clonic seizures. The ED_50_ values for perampanel and diazepam protection against tonic seizures and lethality are presented in Table 5.

**Table 3:** Effects of post-treatment with perampanel and diazepam i.p. 5 min after TETS (0.2 mg/kg, i.p.) on the incidence of clonic and tonic seizures, tonic seizure severity, and the incidence of lethality during a 1 h observation period

Treatment (dose)	*N*	Clonic seizures after treatment	Tonic seizures	Mean tonic seizure severity	Dead 1 h
Vehicle	16	100%	100%	3	100%
Perampanel (0.5 mg/kg)	7	100%	100%	3	85.7%
Perampanel (1 mg/kg)	16	100%	100%	2.7	75%
Perampanel (2 mg/kg)	16	100%	62.5%	1.9	62.5%
Perampanel (3.5 mg/kg)	7	85.7%	57.1%	1.7	42.9%
Perampanel (4 mg/kg)	16	100%	50%	1.4	31.3%
Perampanel (5 mg/kg)	8	100%	12.5%	0.3	0%
Perampanel (6 mg/kg)	8	87.5%	12.5%	0.4	12.5%
Diazepam (0.5 mg/kg)	8	100%	100%	3	87.5%
Diazepam (0.65 mg/kg)	14	100%	78.6%	2.4	78.6%
Diazepam (0.75 mg/kg)	8	87.5%	37.5%	1.1	37.5%
Diazepam (1 mg/kg)	9	88.9%	22.2%	0.7	11.1%
Diazepam (2 mg/kg)	9	44%	11.1%	0.3	11.1%
Diazepam (4 mg/kg)	8	0%	0%	0	0%

### Effect of post-treatment with perampanel or diazepam on oral TETS poisoning

**Fig. 4:**
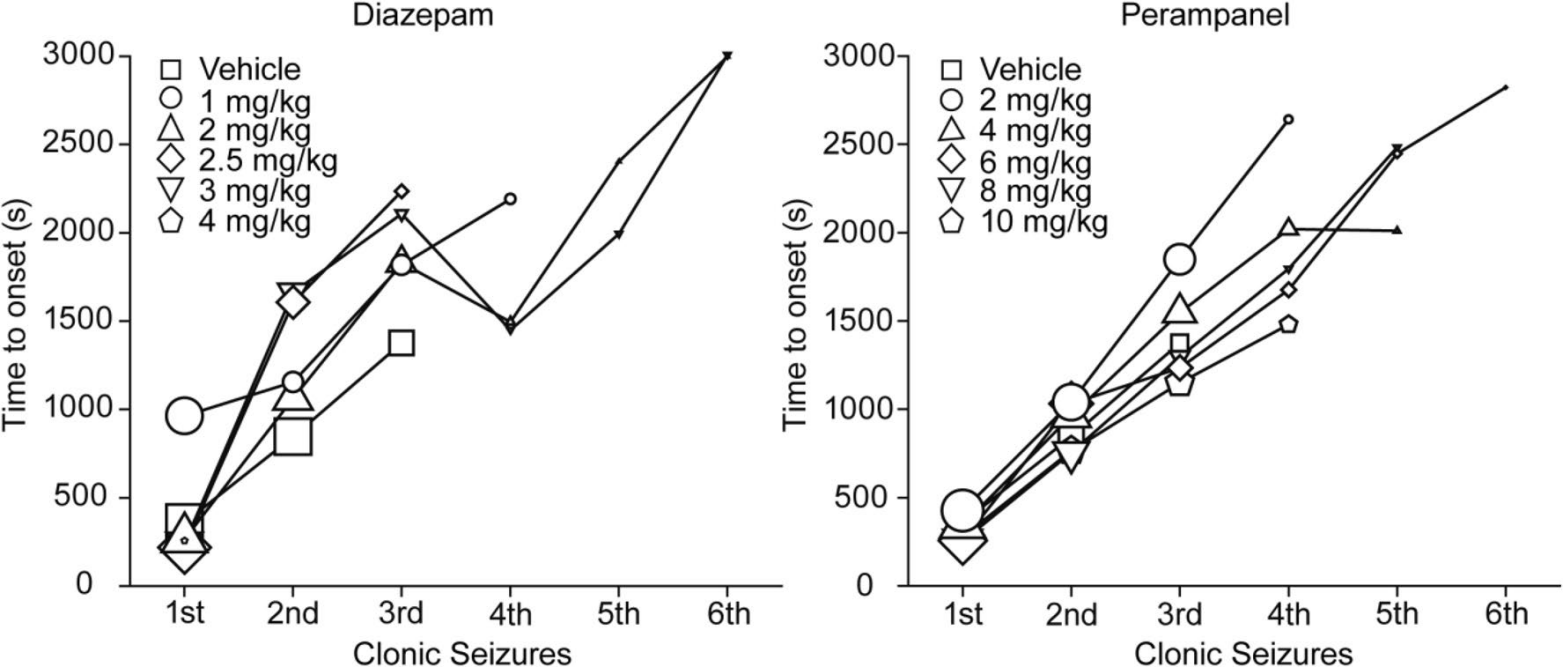
Time to occurrence of clonic seizures in mice treated with vehicle or various doses of either diazepam or perampanel administered 5 min after a 0.4 mg/kg, p.o., dose of TETS. Increasing doses of the treatments generally reduce the incidence and severity of tonic seizures, and improve survival. However, the occurrence of clonic seizures is more resistant. Data points indicate mean time to onset (in seconds) of the *n*th clonic seizure during the 3600 s period following administration of the treatment, which was 5 min after oral gavage of TETS. The size of the symbols represents the fraction of animals experiencing the *n*th clonic seizure in relation to the total number in the dose group

Ingestion, either accidental or intentional, is the most common route of poisoning by TETS. We previously found that the ED_50_ for induction of tonic seizures and lethality for orally administered TETS in mice is 0.22 mg/kg (Zolkowska et al. 2012). In the present study, we sought to determine the efficacy of perampanel and diazepam administered following oral exposure to TETS and delivered a 0.4 mg/kg dose by oral gavage, which was sufficient to induce tonic seizures and lethality in nearly all animals in the absence of treatment. At this dose, clonic seizures began on average ~ 5 min after dosing and tonic seizures and lethality occurred at ~ 20 min. In some animals, tonic seizures occurred earlier than the 5 min treatment time; these animals were excluded from the analysis. Perampanel at doses of 2 mg/kg to 6 mg/kg reduced the incidence and severity of tonic seizures and conferred partial protection against lethality (Table 4). Increasing the dose of perampanel to 8 mg/kg and 10 mg/kg did not provide further protection; therefore, we did not calculate ED_50_ values. Diazepam at high doses (2.5 mg/kg to 4 mg/kg) was somewhat more effective but still did not confer complete protection. During the interval prior to treatment, some animals did not exhibit seizure signs whereas others exhibited myoclonic twitches and up to one clonic seizure. Figure 4 shows the time of occurrence of clonic seizures following each treatment. It is apparent that surviving animals exhibit clonic seizures at similar rates irrespective of the treatment. The results presented in Fig. 4 are representative of the animals in other experiments with TETS (presented in Tables 1, 2, 3) in which clonic seizures were not prevented by the treatments.

**Table 4:** Effects of post-treatment with perampanel and diazepam i.p. 5 min after oral administration of TETS (0.4 mg/kg) on the incidence of clonic and tonic seizures, tonic seizure severity, and the incidence of lethality at 1 h and 24 h

Treatment (dose)	*N*	Clonic Seizures after treatment	Tonic seizures	Mean tonic seizure severity	Dead 1 h	Dead or unsalvageable 24 h
Vehicle	15	100%	100%	3.0	100%	100%
Perampanel (2 mg/kg)	16	100%	75%	2.0	43.8%	100%
Perampanel (4 mg/kg)	16	87.5%	56%	1.6	44%	75%
Perampanel (6 mg/kg)	24	95.8%	41.7%	1.1	33.3%	58.3%
Perampanel (8 mg/kg)	15	80%	53%	1.5	47%	47%
Perampanel (10 mg/kg)	7	85.7%	57.1%	1.6	42.9%	85.7%
Diazepam (1 mg/kg)	7	85.7%	71%	2.1	71.4%	71.4%
Diazepam (2 mg/kg)	15	93.3%	80%	2.4	66.7%	66.7%
Diazepam (2.5 mg/kg)	8	75%	12.5%	0.38	12.5%	12.5%
Diazepam (3 mg/kg)	8	75%	12.5%	0.38	0%	12.5%
Diazepam (4 mg/kg)	7	14.3%	14.3%	0.43	14.3%	14.3%

## Discussion

Perampanel, a potent noncompetitive AMPA receptor antagonist, was first registered for human use in 2012 in the United States and Europe, and is currently approved as a treatment for focal-onset seizures and primary generalized tonic–clonic seizures (Rogawski 2017). The purpose of the present study was to characterize the anti-seizure efficacy of perampanel in models of acute TETS-induced poisoning in mice. For comparison, we conducted a parallel series of experiments with diazepam, a GABA_A_ receptor positive allosteric modulator that is considered a first-line standard-of-care treatment for acute seizures (Greenfield et al. 2021). We used a low (0.14 mg/kg) and a higher (0.2 mg/kg) dose of TETS administered parenterally and a single high dose (0.4 mg/kg) administered by oral gavage. The treatments were administered prior to TETS (pre-treatment) and also at intervals following TETS (post-treatment).

**Table 5:** Summary of ED_50_ values for activity in MES test, motor impairment test, and for protection against TETS-induced tonic seizures and lethality

Test	ED_50_ ± SEM, mg/kg [95% CI]		
		Perampanel	Diazepam
MES		1.50 ± 0.14 [1.22–1.78]	
Horizontal Screen		1.19 ± 0.15 [0.89–1.48]	
TETS (0.2 mg/kg, i.p.), pre-treatment	Tonic seizures	2.19 ± 0.19 [1.81–2.56]	2.15 ± 0.19 [1.79–2.51]
	Lethality at 1 h	1.63 ± 0.18 [1.27–1.98]	2.12 ± 0.23 [1.68–2.57]
TETS (0.14 mg/kg, i.p.), post-treatment (15 min)	Tonic seizures	0.44 ± 0.08 [0.28–0.60]	0.21 ± 0.03 [0.15–0.27]
	Lethality at 24 h	0.34 ± 0.08 [0.19–0.49]	0.32 ± 0.05 [0.23–0.42]
TETS (0.2 mg/kg, i.p.), post-treatment (5 min)	Tonic seizures	3.21 ± 0.33 [2.57–3.84]	0.86 ± 0.10 [0.67–1.06]
	Lethality at 1 h	2.18 ± 0.30 [1.60–2.76]	0.78 ± 0.13 [0.54–1.03]
TETS (0.4 mg/kg, p.o.), post-treatment (5 min)	Tonic seizures	Maximum protection: 6–8	2.02 ± 0.26 [1.51–2.54]
	Lethality at 24 h		1.85 ± 0.27 [1.33–2.37]

In preliminary experiments, we used the MES test to verify the anti-seizure efficacy of perampanel and characterize its time course of action. We found that parenteral perampanel at doses of 1 and 2 mg/kg conferred peak protection at 10 min and that protection lasted up to 30 min. In a subsequent dose–response study with a 10 min pre-treatment interval, the ED_50_ value for seizure protection was 1.50 mg/kg. Our results are consistent with two previous reports. Perampanel in male ddY and male CD1 mice provided protection in the MES seizure test with ED_50_ values of 1.6 mg/kg, p.o., and 1.7 mg/kg, i.p., respectively (Hanada et al. 2011; Zwart et al. 2014). It has been previously reported that AMPA receptor antagonists induce motor impairment at doses similar to those that are protective in the MES test (Yamaguchi et al. 1993). A similar lack of separation between the doses conferring seizure protection and motor incoordination was observed in the previous studies with perampanel in mice (Hanada et al. 2011; Zwart et al. 2014). Consistent with these prior results, in our study, perampanel also caused motor impairment at doses in the same range as those that conferred seizure protection (ED_50_, 1.19 mg/kg, i.p.). Nevertheless, perampanel has acceptable tolerability when used in the treatment of epilepsy indicating that the lack of therapeutic window in rodents does not predict lack of clinical utility. However, perampanel does exhibit a narrow therapeutic window in humans and treatment-emergent side effects, such as dizziness, somnolence, fatigue and gait instability, are common (Rogawski 2017).

In addition to its activity in the MES test, pre-treatment with perampanel protects against seizures in mice induced by the GABA_A_ receptor blocker pentylenetetrazol (Hanada et al. 2011). Similarly, we found that pre-treatment with perampanel protected against seizures and lethality induced by TETS, which is also a GABA_A_ receptor antagonist (Pressly et al. 2021). The potencies for protection against TETS-induced lethality and the reported activity in the PTZ mouse model (0.94 mg/kg, p.o.; Hanada et al. 2011) were comparable. As an antidote for poisoning, post-treatment is required. We studied post-treatment following a lower dose of TETS (0.14 mg/kg) that provided a 15 min window during which the treatment drug could be administered and a higher dose (0.2 mg/kg) that was rapidly lethal requiring the treatment drug to be administered earlier. A 5 min post-treatment interval permitted all animals to receive the treatment drugs with this higher dose. Relatively low doses of either perampanel or diazepam prevented tonic seizures and lethality when administered after the lower dose of TETS. Effective doses of perampanel were substantially lower than those that caused motor impairment. These results indicate that perampanel, and perhaps AMPA receptor antagonists in general, are potentially useful treatments following low-level exposures to TETS. Diazepam was also highly potent at preventing lethality caused by the lower dose of TETS. With a higher dose of TETS that is more rapidly lethal, a larger perampanel dose was required to protect against tonic seizures and lethality. Such higher doses are associated with motor impairment but depending upon the circumstances, a degree of transitory neurological impairment may be acceptable, since the toxicant is lethal. It is noteworthy that perampanel even at very high doses did not cause lethality and animals recovered fully from the neurological impairment. Diazepam was also protective against the higher dose of TETS and as in the case of perampanel, a greater dose was required. However, a relatively smaller increment in diazepam dose was required to protect against the higher TETS dose than was the case for perampanel. Despite the ability of perampanel and diazepam to protect against tonic seizures and lethality, neither of the treatments impacted myoclonic body twitches, and in most situations, even high doses of diazepam only partially suppressed clonic seizures. Therefore, neither of the treatments eliminated all seizure components and can be considered a fully adequate treatment.

Our results are generally in agreement with a previous study of diazepam in the treatment of seizures induced by a high (0.4 mg/kg, i.p.) dose of TETS in mice (Shakarjian et al. 2012). In this prior study, pre-treatment or post-treatment with 1 mg/kg diazepam provided partial protection against tonic seizures and lethality whereas a higher 5 mg/kg dose was more effective. In rats, Moffett et al. (2019) studied post-treatment with even higher doses of diazepam (12.5 and 25 mg/kg), which were found to partially protect against lethality produced by a high (0.6 mg/kg) oral dose of TETS. The benzodiazepines midazolam and lorazepam also enhanced survival. In our study, neither perampanel nor diazepam was fully protective when a high dose of TETS (0.4 mg/kg) was administered orally, although diazepam performed better than perampanel. Consistent with our observation that diazepam minimally suppresses clonic seizure activity, Shakarjian et al. (2012) found that TETS-induced ictal electroencephalogram (EEG) activity corresponding with clonic motor seizures was not suppressed by diazepam. Despite the similar outcomes, certain experimental differences between our study and that of Shakarjian et al. (2012) are worthy of note. The prior study used C57BL/6 mice and, in preliminary testing, we found these mice to be somewhat less sensitive to TETS than the NIH Swiss mice used here (unpublished). An additional difference is that in the prior study, the treatment was administered after the first clonic seizure whereas we administered the treatment at a fixed time after TETS dosing, which we considered to be a more rigorous test.

Since TETS is a GABA_A_ receptor antagonist, it is not unexpected that diazepam, a positive allosteric modulator of GABA_A_ receptors, would confer protection against some seizure manifestations. The effective doses of diazepam that we found to protect against TETS-induced tonic seizures and lethality were similar to the doses that protect against seizures induced by other GABA_A_ receptor antagonists in mice, including pentylenetetrazol, picrotoxin and bicuculline (Swinyard and Castellion 1966; Shenoy et al. 1982). Our results demonstrate that the AMPA receptor antagonist perampanel is also effective against TETS-induced tonic seizures and lethality. AMPA receptors are the main mediators of fast excitatory synaptic transmission in the central nervous system (Rogawski 2013). Perampanel potently inhibits AMPA receptors, resulting in reduced excitatory neurotransmission in local circuits and also attenuated spread of excitation to adjacent and distant sites. This anti-seizure action is entirely distinct from that produced by drugs that act on GABA_A_ receptors. However, AMPA receptor antagonists have broad-spectrum anti-seizure actions, which we have now shown extends to TETS. Despite their distinct mechanisms of action, both perampanel and diazepam are effective in the treatment of tonic seizures and lethality caused by moderate doses of TETS. However, with a high dose (0.4 mg/kg), perampanel failed to protect all animals even at very high doses. Perampanel and diazepam could potentially have some utility in the treatment of TETS-induced seizures but neither drug is effective in eliminating all seizure manifestations.

## Data Availability

Data files are available on request.

## Code availability

Statistical analysis software and documentation are available at: http://mr.ucdavis.edu/laboratory.html.
